# Identification and Characterization of New Resistance-Conferring SGI1s (*Salmonella* Genomic Island 1) in *Proteus mirabilis*

**DOI:** 10.3389/fmicb.2018.03172

**Published:** 2018-12-19

**Authors:** Luyao Bie, Meng Fang, Zhiqiang Li, Mingyu Wang, Hai Xu

**Affiliations:** ^1^State Key Laboratory of Microbial Technology, Microbial Technology Institute, Shandong University, Qingdao, China; ^2^Advanced Research Center for Optics, Shandong University, Qingdao, China

**Keywords:** *Proteus mirabilis*, *Salmonella* genomic island 1, antibiotic resistance, heavy metal resistance, horizontal gene transfer, mobile genetic element

## Abstract

*Salmonella* genomic island 1 (SGI1) is a resistance-conferring chromosomal genomic island that contains an antibiotic resistance gene cluster. The international spread of SGI1-containing strains drew attention to the role of genomic islands in the dissemination of antibiotic resistance genes in *Salmonella* and other Gram-negative bacteria. In this study, five SGI1 variants conferring multidrug and heavy metal resistance were identified and characterized in *Proteus mirabilis* strains: SGI1-*Pm*CAU, SGI1-*Pm*ABB, SGI1-*Pm*JN16, SGI1-*Pm*JN40, and SGI1-*Pm*JN48. The genetic structures of SGI1-*Pm*CAU and SGI1-*Pm*ABB were identical to previously reported SGI1s, while structural analysis showed that SGI1-*Pm*JN16, SGI1-*Pm*JN40, and SGI1-*Pm*JN48 are new SGI1 variants. SGI1-*Pm*JN16 is derived from SGI1-Z with the MDR region containing a new gene cassette array *dfrA12-orfF-aadA2-qacE*Δ*1-sul1-chrA-orf1*. SGI1-*Pm*JN40 has an unprecedented structure that contains two right direct repeat sequences separated by a transcriptional regulator-rich DNA fragment, and is predicted to form two different extrachromosomal mobilizable DNA circles for dissemination. SGI1-*Pm*JN48 lacks a common ORF S044, and its right junction region exhibits a unique genetic organization due to the reverse integration of a *P. mirabilis* chromosomal gene cluster and the insertion of part of a *P. mirabilis* plasmid, making it the largest known SGI1 to date (189.1 kb). Further mobility functional analysis suggested that these SGIs can be excised from the chromosome for transfer between bacteria, which promotes the horizontal transfer of antibiotic and heavy metal resistance genes. The identification and characterization of the new SGI1 variants in this work suggested the diversity of SGI1 structures and their significant roles in the evolution of bacteria.

## Introduction

*Salmonella* genomic island 1 (SGI1) is a genomic island containing an antibiotic resistance gene cluster initially identified in *Salmonella enterica* serovar Typhimurium phage type DT104 strain (Boyd et al., 2001). The multidrug resistance (MDR) region in SGI1 is a complex In4-type class 1 integron, which contains five antibiotic resistance genes conferring resistance to ampicillin (*bla*_PSE−1_), chloramphenicol and florfenicol (*floR*), streptomycin and spectinomycin (*aadA2*), sulfonamides (*sul1*), and tetracycline [*tet(G)*] (Boyd et al., 2001). Since the identification of SGI1 in *S.* *enterica* Typhimurium DT104, SGI1 variants with high genetic diversities on the MDR regions and backbones were found in other *S. enterica* serovars (Levings et al., 2005), *Proteus mirabilis* (Ahmed et al., 2006), and *Morganella morganii* (Schultz et al., 2017). From a classification perspective, based on differences of antibiotic resistance gene clusters located at MDR regions, SGI1 was classified from SGI1-A to SGI1-Z (Boyd et al., 2002, 2008; Levings et al., 2007; Doublet et al., 2009; Bi et al., 2011; Le Hello et al., 2012; Lei et al., 2014, 2015). Additionally, six SGI variants (SGI1-B, SGI1-K, SGI1-P, SGI1-Q, SGI1-L, and SGI1-J) were subdivided into two or more subgroups (Levings et al., 2005, 2008; Doublet et al., 2008, 2009; Chu et al., 2012; Lei et al., 2015).

In recent years, three SGI1-related elements, SGI2, *Proteus* genomic island (PGI1/PGI2) and *Acinetobacter* genomic island 1 (AGI1), were reported in *S. enterica*, *P. mirabilis* and *Acinetobacter baumannii*, respectively (Levings et al., 2008; Siebor and Neuwirth, 2014; Hamidian et al., 2015; Lei et al., 2018). All these four genomic islands integrate into the 3′-end of chromosomal *trmE* gene and carry diverse antibiotic resistance genes in their MDR regions (Hall, 2010; Hamidian et al., 2015; Lei et al., 2018). Of particular interest, SGI1-V with extended-spectrum β-lactamase (ESBL) gene (*bla*_VEB−6_) and PGI1-*Pm*PEL carrying carbapenemase gene (*bla*_NDM−1_) in MDR region were detected in clinical *P. mirabilis* strains (Siebor and Neuwirth, 2011; Girlich et al., 2015). The emergence of these strains carrying SGI1/PGI1 with ESBL gene and/or carbapenemase gene is of great concern to public health, as β-lactams and carbapenems remain the most widely used antibiotics for the treatment of bacterial infection.

It has been reported that the transcriptional activator complex AcaCD, whose coding genes are carried by IncA/C type plasmids, triggers the excision and conjugative transfer of SGI1/PGI1 (Kiss et al., 2015). SGI1/PGI1 could further be mobilized and transferred into a broad range of Enterobacteriaceae with the help of conjugative IncA/C plasmids (Siebor et al., 2016), suggesting that SGI1s/PGI1s could act as mobilizable elements for the dissemination of resistance genes. The transfer of these MDR genomic islands (GIs) increased the level of antimicrobial resistance among Enterobacteriaceae, and the GI-facilitated horizontal gene transfer contributed to the diversification and adaptation of microorganisms, therefore having an impact on the genome plasticity and evolution of bacteria (Juhas et al., 2009).

*Proteus mirabilis* has been recognized to be the causative agent of a variety of opportunistic nosocomial infections, and it is especially associated with urinary tract infections. It has become a potential public health concern in recent years (Cohn et al., 2003). Since the detection of a SGI1 variant in *P. mirabilis* in 2006, the number of reported SGI1 variants in *P. mirabilis* isolates from clinical, animal, or food in China and France has been increasing (such as SGI1-O, SGI1U-Z, SGI1-B2, SGI1-*Pm*BRI, SGI1-*Pm*CAU, and SGI1-*Pm*ABB) (Boyd et al., 2008; Bi et al., 2011; Siebor and Neuwirth, 2013; Lei et al., 2014, 2015; Girlich et al., 2015; Qin et al., 2015). These findings lead to a strong suggestion that *P. mirabilis* may serve as a host for mobilizable genetic elements and facilitates the dissemination of antimicrobial resistance.

In the current study, new SGI1 variants were characterized in multidrug resistant *P. mirabilis* strains and their genetic structures were mapped. These findings improve our understanding of the diversity of SGI1 structures and the prevalence of antibiotic resistant genomic island in *P. mirabilis*.

**FIGURE 1:**
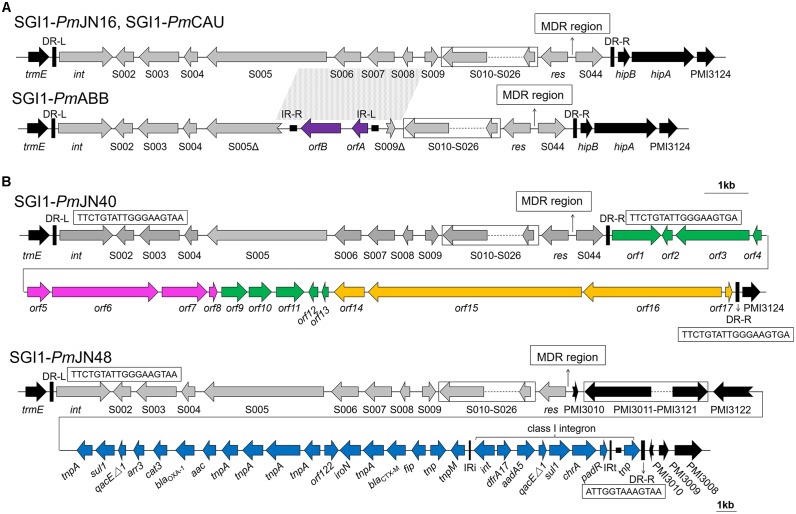
Backbone structures of SGI1s identified in this work. **(A)** Backbone structures of SGI1-*Pm*CAU, SGI1-*Pm*ABB, and SGI1-*Pm*JN16. **(B)** Backbone structures of SGI1-*Pm*JN40 and SGI1-*Pm*JN48. SGI1 backbones are shown with different colored arrows based on their origins: SGI1 conserved backbone (gray), IS*1356* (purple), chromosomal DNA of *Vibrio parahaemolyticus* (green), chromosomal DNA of *Shewanella bicestrii* (pink), chromosomal DNA of *Photobacterium damselae* (orange), p*Pm*14C18 (blue), and chromosomal DNA of *Proteus mirabilis* (black). The direct repeats of SGI1 (DR-L/DR-R) and inverted repeats of class I integron (IRi/IRt) are indicated by thick vertical bars and thin vertical bars, respectively. The small filled squares represent inverted repeats of the IS. The position of the MDR regions is indicated by thin arrows. Truncated genes are shown by swallowtail arrows.

## Materials and Methods

### Sampling and Bacterial Isolation

During the time period from June to September 2013, fifty-seven antibiotic-resistant *P. mirabilis* strains were isolated from a commercial broiler slaughter plant in Shandong Province, China. Sterile, moistened swabs were used to wipe 2 cm^2^ areas from the surfaces and insides of 107 eviscerated broiler chicken carcasses by the method of Wu et al. (2015). Swabs were agitated in 10 mL of sterile saline (0.9% NaCl), and the suspensions were serially diluted. A 100 μL aliquot of each dilution was spread onto eosin methylene blue (EMB) plates (AOBOX, Beijing, China) that were supplemented with one of the following six antimicrobial agents. Antibiotics used for screening these strains included amoxicillin–clavulanic acid (32/16 mg/L), ciprofloxacin (4 mg/L), amikacin (32 mg/L), doxycycline (16 mg/L), sulbactam–cefoperazone (64/64 mg/L), and sulfamethoxazole–trimethoprim (4/76 mg/L). The isolated strains were resistant to at least one of the antibiotics used. Genomic DNA was extracted from the bacteria using bacteria genome extraction kit (GeneRay, Shanghai, China).

### Plasmid

The IncA/C type pR55 plasmid capable of mediating conjugative transfer of SGI1s is a kind gift from Prof. Hongning Wang from Sichuan University (Douard et al., 2010; Doublet et al., 2012). The plasmid is harbored by an *Escherichia coli* C600 strain.

### Antimicrobial Susceptibility Assay

The antimicrobial sensitivity phenotypes of *P. mirabilis* strains were determined by the disk diffusion method using Müller–Hinton agar plates following CLSI guidelines M100-S26 (CLSI, 2016). The following antimicrobial agents were used: ceftazidime (CAZ, 30 mg/L), ampicillin (AMP, 10 mg/L), cefotaxime (CTX, 30 mg/L), nalidixic acid (NAL, 30 mg/L), streptomycin (STR, 10 mg/L), kanamycin (KAN, 30 mg/L), tetracycline (TET, 30 mg/L), trimethoprim (TMP, 5 mg/L), chloramphenicol (CHL, 30 mg/L), sulfisoxazole (SFX, 250 mg/L), erythromycin (ERY, 15 mg/L), rifampin (RIF, 5 mg/L), and imipenem (IPM, 10 mg/L). *E. coli* ATCC 25922 was used as a quality control.

### Detection and Sequencing of SGI1s

*Salmonella* genomic island 1 and its chromosomal location were detected using primers targeting the left junction (primer pairs PmLJ1/LJ-R1), right junction (primer pairs 104-RJ/PmRJ1), and ORF S026 (primer pairs S026-F/S026-R) in the chromosome. The structures of SGI1s were analyzed by PCR-mapping and sequencing DNA fragments using the corresponding primers described in Supplementary Table S1.

The special right junction regions of SGI1-*Pm*JN40 and SGI1-*Pm*JN48 were further obtained by genome sequencing of SGI1-containing *P. mirabilis* strains using Illumina MiSeq (Illumina Inc., San Diego, CA, United States) sequencing platform with a 400-bp paired-end library, as well as SOAPdevono v2.04 and GapCloser v1.12 software to construct *de novo* assemblies (Luo et al., 2012). Gaps between scaffolds were closed by PCR followed by sequencing.

### Bioinformatics

The complete nucleotide sequences of SGI1s were analyzed using the BLAST algorithm^1^. The GenBank accession numbers for SGI1-*Pm*CAU, SGI1-*Pm*ABB, SGI1*-Pm*JN40, SGI1-*Pm*JN16, and SGI1-*Pm*JN48 are JX089581.1 (Siebor and Neuwirth, 2013), JX121638.1 (Siebor and Neuwirth, 2013), MF576128, MF576129, and MF576130, respectively.

### Chromate Resistance Assays

Chromate resistance was measured by the comparison of growth rates in LB media. Overnight cultures were diluted 2000-fold into 50-mL flasks containing 25 mL of fresh medium supplemented with different chromate (K_2_CrO_4_) concentrations. The bacterial suspensions were incubated at 37°C with shaking at 170 rpm for 12 h, and the value of OD_600_ was measured (Alvarez et al., 1999; Branco et al., 2008).

### Stability Test for SGI1

Ten microliter cultures of all the five SGI1-positive *P. mirabilis* strains were serially transferred into 3 mL LB medium (300 × dilution per passage), grown at 37°C under vigorous shaking without antibiotic selection. A total of 24 passages over 192 h were performed for stability test. Cultures from the final passage were plated on LB agar plates without antibiotic selection, and one hundred clones were picked from each strain for the detection of presence/absence of SGI1 by PCR reactions.

### Conjugative Transfer Test for SGI1

The pR55 helper plasmid harbored by *E. coli* C600 was transferred to SGI1-containing *P. mirabilis* via conjugation prior to testing the transferability of SGI1s identified in this work. Briefly, pR55-containing *E. coli* C600 was grown to late-log phase, and mixed together with the recipient SGI1-carrying *P. mirabilis* strains at a ratio of 4:1. The mixture was subsequently incubated at 37°C overnight without shaking, diluted 10-fold, and plated on selective MacConkey agar plates containing chloramphenicol (30 mg/L) and trimethoprim (30 mg/L).

To test whether SGI1s found in this work can be mobilized to another bacterium via conjugative transfer, we mixed pR55+SGI1-containing *P. mirabilis* donor strains with the sodium azide-resistant *E. coli* recipient strain J53 following procedures described above. Sodium azide (200 mg/L) and trimethoprim (30 mg/L)/streptomycin (30 mg/L) were used to select transconjugants. The SGI1 frequency of transfer was determined by dividing the number of *E. coli* SGI1 transconjugants by the number of *P. mirabilis* donor cells, as previously described (Douard et al., 2010).

The transconjugants were examined for the presence of SGI1-specific genes with PCR reactions, in order to confirm the conjugative transfer of SGI1s from respective *P. mirabilis* donor strains to the recipient *E. coli* J53 strain. The primers used for screening SGI1-positive transconjugants are listed in Supplementary Table S1.

## Results

### Detection of SGI1s in Multidrug Resistant *P. mirabilis*

The general structure and mechanism of site-specific integration for SGI1 in *P. mirabilis* can be depicted in a model shown in Supplementary Figure S1. The left junction region of SGI1s as well as ORF S026 were detected in 5 out of 57 multidrug resistant *P. mirabilis* strains (JN16, JN29, JN40, JN47, and JN48) isolated from a commercial broiler slaughterhouse. Interestingly, *P. mirabilis* JN40 and JN48 were negative for the right junction with primers 104-RJ (S044 specific) and PmRJ1 (*hipB* specific) (Supplementary Figure S2). The right junction regions of these two SGI1s were then further analyzed by PCR with primer pairs 104-RJ/hipA-R1 (*hipA* specific) and 104-RJ/MP-R1 (specific for the membrane protein PMI3124-encoding gene) that respectively, target the two genes downstream of *hipB* on the chromosome of *P. mirabilis* HI4320 (Siebor and Neuwirth, 2013). However, the right junction regions were still undetected in *P. mirabilis* JN40 and JN48 strains using the above primers (Supplementary Figure S2). These results suggested a different right junction structure of SGI1s in *P. mirabilis* JN40 and JN48 in comparison with the conventional right junction region.

**Table 1:** SGI1-containing *Proteus mirabilis* isolates characterized in this study.

Strain	Antimicrobial resistance profile	Containing SGI1	SGI1 size (kb)	Integron gene cassette (s)	Other resistance gene (s)
JN16	**SFX**, AMP, CTX, NAL, **STR**, **KAN**, TET, **TMP**, CHL	SGI1-*Pm*JN16	34.8	*dfrA12-orfF-aad2 chrA*	*sul1*
JN29	**SFX**, AMP, CTX, NAL, STR, KAN, TET, **TMP**, CHL	SGI1-*Pm*CAU	33.2	*dfrA1-orfC*	*sul1*
JN40	**SFX**, CAZ, AMP, CTX, NAL, **STR**, **KAN**, TET, TMP, CHL	SGI1-*Pm*JN40	56.7	*aadA2*	*sul1*
JN47	**SFX**, AMP, CTX, NAL, **STR**, **KAN**, TET, TMP, CHL	SGI1-*Pm*ABB	32.0	*aacCA5-aadA7*	*sul1*
JN48	**SFX**, **AMP**, **CTX**, NAL, **STR**, **KAN**, TET, **TMP**, **CHL**, **ERY**, **RIF**	SGI1-*Pm*JN48	189.1	*bla*_PSE−1_, *dfrA17-aadA5, chrA*	*mphA-mrx-mphR*, *arr3-cat3-bla*_OXA−1_- *aac*, *bla*_CTX−M_, *sul1*

*CAZ, ceftazidime; AMP, ampicillin; CTX, cefotaxime; NAL, nalidixic acid; STR, streptomycin; KAN, kanamycin; TET, tetracycline; TMP, trimethoprim; CHL, chloramphenicol; SFX, sulfisoxazole; ERY, erythromycin; RIF, rifampin. The resistance phenotypes corresponding to drug resistance genes on SGI1 were indicated in bold font.*

### Sequencing of SGI1 Variants

The complete sequences of SGI1s in *P. mirabilis* JN16, JN29, and JN47 were obtained by PCR mapping and sequencing of the backbones and MDR regions using primers listed in Supplementary Table S1. For SGI1s containing unconventional right junction structures, whole genome sequencing of their host strains (*P. mirabilis* JN40 and JN48) was performed to obtain their full length sequences. Complete nucleotide sequences of SGI1s were analyzed using the BLAST algorism^1^. The genetic organization of SGI1 in *P. mirabilis* JN29 and JN47 was identical to the previously reported SGI1-*Pm*CAU and SGI1-*Pm*ABB, respectively (Siebor and Neuwirth, 2013), while SGI1s identified in *P. mirabilis* JN16, JN40, and JN48 were not previously observed (Table 1). According to the nomenclature system of SGI1 (Mulvey et al., 2006), these three new SGI1s were subsequently denominated SGI1-*Pm*JN16, SGI1-*Pm*JN40, and SGI1-*Pm*JN48.

### Characterization of the SGI1 Backbones

The structures of the SGI1 backbones are displayed in Figure 1. Sequence analysis of SGI1-*Pm*CAU, SGI1-*Pm*ABB, SGI1- *Pm*JN16, and SGI1-*Pm*JN40 showed that their left direct repeat (DR-L) was nearly identical to the right direct repeat (DR-R). As shown in Figure 1A, SGI1-*Pm*CAU and SGI1-*Pm*JN16 contain the conventional backbone regions (S001–S027 and S044) as previously reported (Lei et al., 2015). In SGI1-*Pm*ABB, the region spanning from ORF S005 to ORF S009 was deleted (2780 bp), and replaced by an insertion of IS*1359* of the IS*3* family initially described in *Vibrio cholera* (GenBank accession number EU664602) (Doublet et al., 2009). SGI1-*Pm*CAU, SGI1-*Pm*ABB and SGI1-*Pm*JN16 integrated between the chromosomal genes *trmE* and *hipB/hipA*, but not for SGI1-*Pm*JN40 and SGI1*Pm*JN48.

SGI1-*Pm*JN40 found in *P. mirabilis* JN40 displays a unique and unprecedented backbone structure that contain one DR-L and two identical DR-Rs (Figure 1B). Present between the two DR-Rs is a 23.7 kb fragment (from *orf1* to *orf17*) that is a combination of *V. parahaemolyticus* (GenBank accession number CP026041.1), *Shewanella bicestrii* (GenBank accession number CP022358.1), and *Photobacterium damselae* (GenBank accession number CP021151.1) genomic DNA (Figure 1B). This DNA fragment contains genes encoding transcriptional regulators (*orf4* and *orf12*), DNA binding proteins (*orf2* and *orf7*) and toxin-antitoxin (*orf5* and *orf8*). The transcriptional regulators encoded by *orf4* and *orf12* belong to AlpA family phage regulatory protein and XRE family transcriptional regulator, respectively. It has been reported that AlpA-type regulators are positive regulatory factors of integrase in the P4-like prophage (Kirby et al., 1994). In addition, The XRE-type regulators were shown to function as a repressor of conjugative transfer and participate in regulation of excision and transfer of genomic island (López-Fuentes et al., 2015). The two DNA binding proteins encoded by SGI1-*Pm*JN40 may function in binding to promoter regions for transcriptional regulation, although they cannot be immediately classified as transcriptional regulators. Furthermore, TA systems contribute to the maintenance of genetic elements by reducing growth, inhibiting growth or killing a subpopulation of cells (Hernández-Arriaga et al., 2014; Marsan et al., 2017). Therefore, it can be inferred that these functional genes may play a potential regulatory role. The presence of two identical DR-Rs in SGI1-*Pm*JN40 suggests its potential to form two different mobilizable DNA species in the form of free circles, respectively, denominated SGI1-*Pm*JN40-S (from *int* to S044) and SGI1-*Pm*JN40-L (from *int* to *orf17*). Considering the potential regulatory role of the genes between two DR-Rs (from *orf1* to *orf17*), the larger circle (SGI1-*Pm*JN40-L) may have a regulatory function.

SGI1-*Pm*JN48 is a new SGI1 variant with a special genetic organization in its right junction region. ORF S044 was absent in SGI1-*Pm*JN48 and replaced by the insertion of a large gene fragment containing *P. mirabilis* chromosomal DNA (PMI3010-PMI3122), followed by a gene cluster identical to part of a *P. mirabilis* plasmid p*Pm*14C18 (GenBank accession number KU605240) (Figure 1B). Considering the existence of an imperfect direct repeat (ATTGGTAAAGTAA) in the downstream of the insertion (Figure 1B), we propose that the insertion from PMI3010 to *padR*/*tnp* was part of SGI1-*Pm*JN48. The size of SGI1-*Pm*JN48 is 189.1 kb, which is the longest SGI1 identified to date.

**Table 2:** Conjugative transfer frequency of *P. mirabilis* SGI1s.

*P. mirabilis* donor strain	SGI1 variant	Conjugative plasmid	SGI1 transfer frequency
JN29	SGI1-*Pm*CAU	IncA/C pR55	2.5 × 10^−5^
JN40	SGI1-*Pm*JN40-L	IncA/C pR55	4.4 × 10^−6^
JN47	SGI1-*Pm*ABB	IncA/C pR55	1.3 × 10^−5^
JN48	SGI1-*Pm*JN48	IncA/C pR55	2.3 × 10^−6^

**FIGURE 2:**
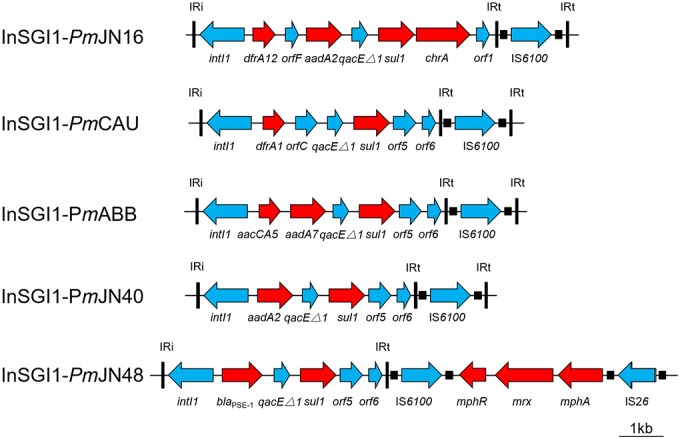
Schematic view of the MDR region within the SGI1 of *P. mirabilis*. Antibiotic resistance genes are indicated by red arrows. Thin vertical bars represent the inverted repeats of class I integron (IRi/IRt). Small filled squares represent the inverted repeats of the IS.

### Characterization of the MDR Regions

The complex genetic structures of MDR regions in five SGI1s were determined. All the MDR regions include a class 1 integron belonging to the In4 family as previously reported (Boyd et al., 2001; Ahmed et al., 2006). InSGI1-*Pm*CAU, InSGI1-*Pm*ABB and InSGI1*-Pm*JN40 contain the *dfrA1-orfC*, *aacCA5-aadA7* and *aadA2* gene cassettes in their variable regions, respectively (Figure 2). The resistance phenotypes mediated by these drug resistance genes of the SGI1-containing bacteria are shown in Table 1.

InSGI1-*Pm*JN16 has a unique MDR structure that has not been observed previously in SGI1. It contains a *dfrA12-orfF-aadA2-qacE*Δ*1-sul1-chrA-orf1* gene cassette. The gene cassette array *dfrA12-orfF-aadA2-qacE*Δ*1-sul1* was similar with the MDR region of SGI1-Z identified in *P. mirabilis* (Qin et al., 2015). However, the *sul1* gene in InSGI1-*Pm*JN16 was followed by the *chrA-orf1* gene cassette developing a new structure of SGI1 MDR. It is noteworthy that the *chrA* gene is expected to confer chromate resistance by encoding a chromate transport protein (Alvarez et al., 1999). In order to confirm this resistance, we tested the chromate resistance of SGI1-*Pm*JN16-positive *P. mirabilis* JN16 using the *chrA*-negative *P. mirabilis* JN49 as the negative control (Supplementary Figure S3). The JN16 strain showed significantly higher chromate tolerance than that in the JN49 strain. This result indicated that *chrA* can indeed encode for chromate resistance.

**FIGURE 3:**
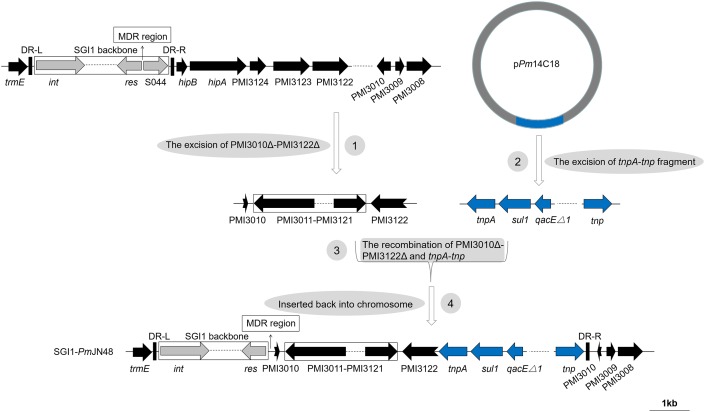
The four-step pathway for the generation of SGI1-*Pm*JN48. SGI1-*Pm*JN48 backbone was shown with different colored arrows: conserved SGI1 backbone (gray), chromosomal DNA of *P. mirabilis* (black), *tnpA-tnp* fragment of plasmid p*Pm*14C18 (blue). The direct repeats of SGI1 (DR-L/DR-R) and the position of the MDR regions are indicated by thick vertical bars and thin arrows, respectively. Truncated genes are shown by swallowtail arrows.

SGI1*Pm*JN48 carries two MDR clusters in its conventional MDR region, including *bla*_PSE−1_-*qacE*Δ*1-sul1* and *mphA-mrx-mphR* (Figure 2). The *bla*_PSE−1_-*qacE*Δ*1-sul1* gene cassette is part of SGI1-B, while the macrolide inactivation gene cluster *mphA-mrx-mphR* conferring resistance to erythromycin has been identified in SGI1-B2 (Hall, 2010; Lei et al., 2015). Therefore, the MDR region of SGI1-*Pm*JN48 might derive from the recombination and rearrangement of SGI1-B and SGI1-B2. In addition to these two MDR clusters, other resistance gene clusters were found in SGI1-*Pm*JN48 that were originally part of the plasmid p*Pm*14C18 (GenBank accession number KU605240): *arr3-cat3-bla*_OXA−1_-*aac*, *bla*_CTX−M_ and part of a class 1 integron (*intI1-dfrA17-aadA5-qacE*Δ*1-sul1-chrA*) (Figure 1B). These genes could confer resistance to rifampin, chloramphenicol, β-lactams, aminoglycosides, sulfonamides, quaternary ammonium compound, and chromate, respectively (Alvarez et al., 1999; Partridge et al., 2009). The chromate resistance phenotype of *P. mirabilis* JN48 that harbors SGI1*Pm*JN48 was described in Supplementary Figure S3, confirming the functionality of the *chrA* gene.

### Mobility and Stability of SGI1

*Salmonella* genomic island 1s can excise from the chromosome, forming a free circle that could be transferred with the help of IncA/C plasmid (Doublet et al., 2005; Douard et al., 2010). The free circular form of SGI1 has been previously identified in *S. enterica* and *P. mirabilis*. The formation of free circle implied the correct excision of SGI1 and the capacity of SGI1 to be transferred by the helper plasmid (Kiss et al., 2015; Lei et al., 2015). In this study, the circular extrachromosomal forms (*attP*) of SGI1-*Pm*CAU, SGI1-*Pm*JN40-S, SGI1-*Pm*ABB and SGI1-*Pm*JN16 were detected by PCR using the circ1/circ2 primers (Supplementary Figures S1, S2), suggesting the mobility of SGI1s.

In order to further confirm the mobility of SGI1s, we performed conjugative transfer tests for SGI1-containing *P. mirabilis* strains that contain the IncA/C-type pR55 helper plasmid. Primers were designed to detect SGI1s in transconjugants (targeted genes: *xis* and S044 for all SGI1s; *orf4*, *orf16*, and *orf17* for SGI1-*Pm*JN40; PMI3015, PMI3046, and *chrA* for SGI1-*Pm*JN48). We were able to detect the conjugative transfer of SGI1-*Pm*CAU, SGI1-*Pm*JN40-L (large circular form of SGI1-*Pm*JN40), SGI1-*Pm*ABB and SGI1-*Pm*JN48 from the donor strains to recipient *E. coli* J53 strain at frequencies between 10^−5^ and 10^−6^, suggesting that these SGI1s are functional and can be transferred between bacterial cells with the help of IncA/C plasmids (Table 2 and Supplementary Figure S4). Unfortunately, we were unable to transfer pR55 to *P. mirabilis* JN16 that harbors SGI1-*Pm*JN16, therefore weren’t able to further characterize the conjugative transferability of SGI1-*Pm*JN16. Nevertheless, the capability of SGI1-*Pm*JN16 to form circular forms suggests its potential to be transferred to another cell.

The stability of SGI1 in the chromosome of *S. enterica* and *P. mirabilis* has been previously described (Kiss et al., 2012; Lei et al., 2015). In order to test the stability of SGI1s found in this study, all five SGI1-containing strains were propagated, lasting for 24 passages and 192 h in the absence of antimicrobial pressure. No SGI1-negative clone was detected from the 500 clones picked (100 clones were picked from each strain) after the final passage, suggesting that SGI1s found in this work are stable in *P. mirabilis*.

The mobility and stability tests performed on the five SGI1s found in this work suggest that they are stable, that they can excise from the chromosome, and that they can transfer between cells. During the transfer of SGI1s, they can serve as vectors of resistance genes and play a significant role in the dissemination of antibiotic and heavy metal resistance.

## Discussion

Five SGI1s were identified and characterized in multidrug resistant *P. mirabilis* strains, and different antibiotic and heavy metal resistant gene clusters were identified in their MDR regions, suggesting their roles in the conferment and dissemination of resistance. Among the five SGI1s, two SGI1s (SGI1-*Pm*CAU and SGI1-*Pm*ABB) are identical to previously identified counterparts, while all remaining three SGI1s (SGI1-*Pm*JN16, SGI1-*Pm*JN40, and SGI1-*Pm*JN48) showed new structures and functions.

SGI1*-Pm*JN16 has a conventional backbone structure and a new MDR region structure (Figures 1, 2). The integron variable region gene cassette array of SGI1-*Pm*JN16 is *dfrA12-orfF-aadA2-qacE*Δ*1-sul1-chrA-orf1* that has not been reported in other SGI1s. This new gene cassette array is a variant of its counterpart in SGI1-Z (*dfrA12-orfF-aadA2-qacE*Δ*1-sul1*). Therefore, we propose that SGI1-*Pm*JN16 is derived from previously reported SGI1-Z (Qin et al., 2015) by adding new functional chromate resistance gene *chrA*, which adds additional resistance feature for this SGI1.

SGI1-*Pm*JN40 is a unique SGI1 that contains two identical DR-Rs. This unprecedented structure suggests its capability to form two mobilizable circular forms via integrase-mediated recombination. The larger SGI1 species (SGI1-*Pm*JN40-L) contains a hybrid DNA fragment of *V. parahaemolyticus*, *S. bicestrii*, and *P. damselae* chromosomal DNA that is rich in regulator-coding genes (*orf2, orf4*, *orf5, orf7*, *orf8*, and *orf12*) and potentially functions in regulation. While only the smaller circular form of SGI1-*Pm*JN40 (SGI1-*Pm*JN40-S) was directly observed, we were able to detect the conjugative transfer of the large circular form of SGI1-*Pm*JN40 (SGI1-*Pm*JN40-L). Therefore, we hypothesize that both forms are concurrently present and functional in *P. mirabilis*.

For the S044-lacking SGI1-*Pm*JN48, a large insertion containing *P. mirabilis* chromosomal DNA (PMI3010-PMI3122 in the inverse orientation) and plasmid-borne gene cluster from p*Pm*14C18 is present (Figure 1B). As shown in Figure 3, a complex four-step event likely took place for the generation of SGI1-*Pm*JN48: PMI3010Δ-PMI3122Δ was excised from the chromosome, recombined with the *tnpA*-*tnp* fragment excised from the plasmid p*Pm*14C18, and inserted back into *P. mirabilis* chromosome by replacing S044*-*PMI3122Δ. SGI1-*Pm*JN48 contains multiple MDR regions (*bla*_PSE−1_-*qacE*Δ*1-sul1*, *mphA-mrx-mphR*, *arr3-cat3-bla*_OXA−1_-*aac*, *bla*_CTX−M_, and *dfrA17-aadA5-qacE*Δ*1-sul1-chrA*) that confer resistance to a series of antibiotics and heavy metal including erythromycin, rifampin, chloramphenicol, ampicillin, trimethoprim, streptomycin/kanamycin, sulfisoxazole, and chromate (Table 1 and Supplementary Figure S3), making it a strong disseminator of MDR. It is noteworthy that these gene clusters are surrounded by different transposase genes of insertion sequences (Figure 1B), leading to the proposal that the insertion of these resistance genes is mediated by various transposons and ISs. Experimental evidence in this work showed that this large SGI1 can be mobilized and transferred between cells via conjugative transfer, suggesting it is the largest functional SGI1 that has a unique structure, to the best of our knowledge.

The newly identified SGIs in this work give us a better understanding of the genetic diversity of SGI1s: SGI1-*Pm*JN40 is a new type SGI1 that can potentially form two circular species for dissemination; SGI1-*Pm*JN48 contains a very long DNA insert that can potentially form a MDR disseminator with the size of over 150 kb. These new structures are drastically different from previously identified SGI1s, and encourage us to expand our search for more unconventional mobile genetic elements.

## Author Contributions

LB and MF performed the experiments. LB, ZL, MW, and HX analyzed the data. LB, MW, and HX wrote the manuscript. ZL critically revised the manuscript. MW and HX conceived of the study. LB, MF, ZL, MW, and HX approved the final manuscript.

## Conflict of Interest Statement

The authors declare that the research was conducted in the absence of any commercial or financial relationships that could be construed as a potential conflict of interest.

## Footnotes

1 http://blast.ncbi.nlm.nih.gov/Blast.cgi
