# Supplementary figures and images for: Identification and Characterization of New Resistance-Conferring SGI1s (Salmonella Genomic Island 1) in Proteus mirabilis
Source: Front Microbiol. 2018 Dec 19;9:3172. doi: 10.3389/fmicb.2018.03172 (PMC6305713; doi:10.3389/fmicb.2018.03172)

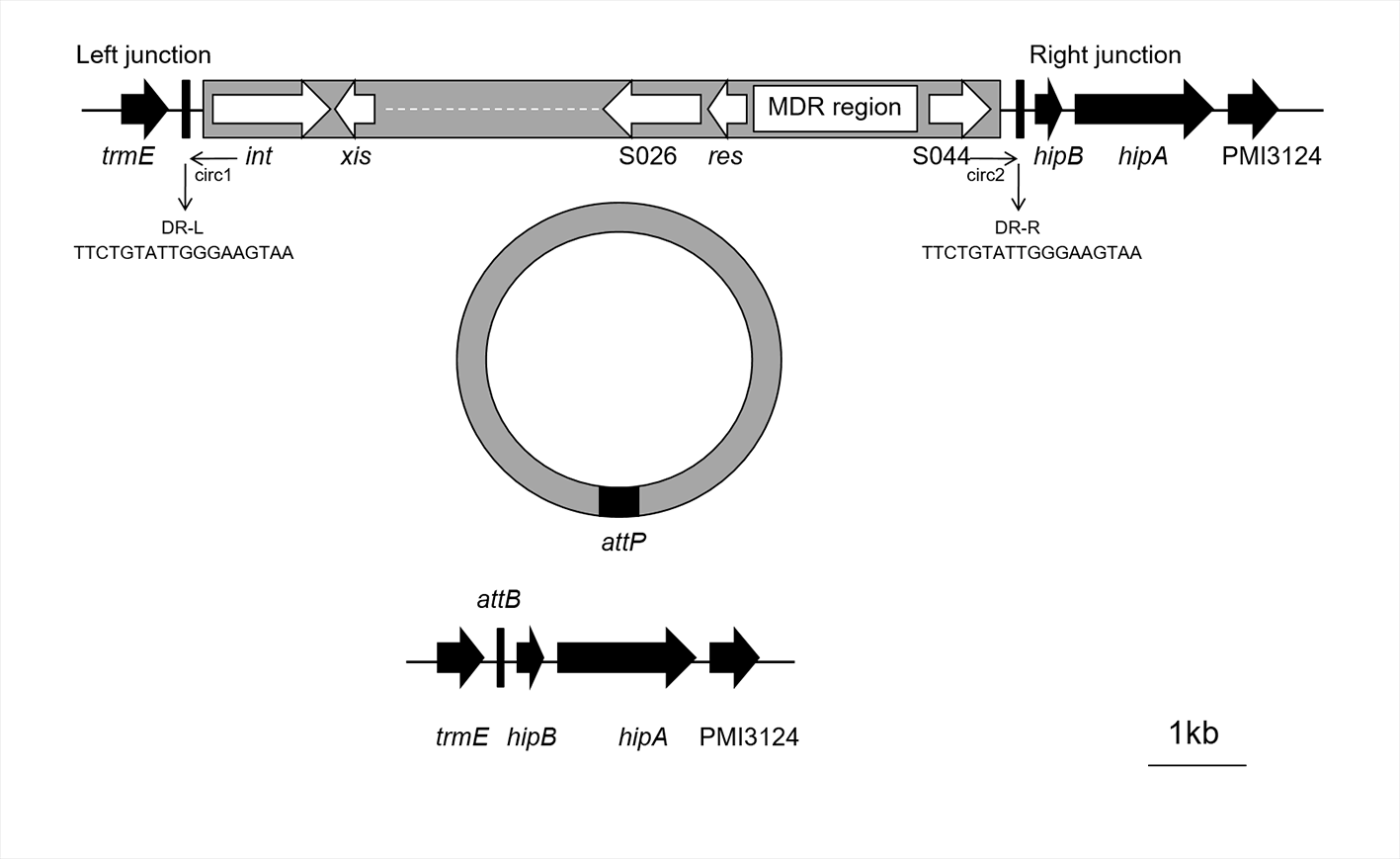

Supplement: Supplementary file 1 [file Image_1.TIF]

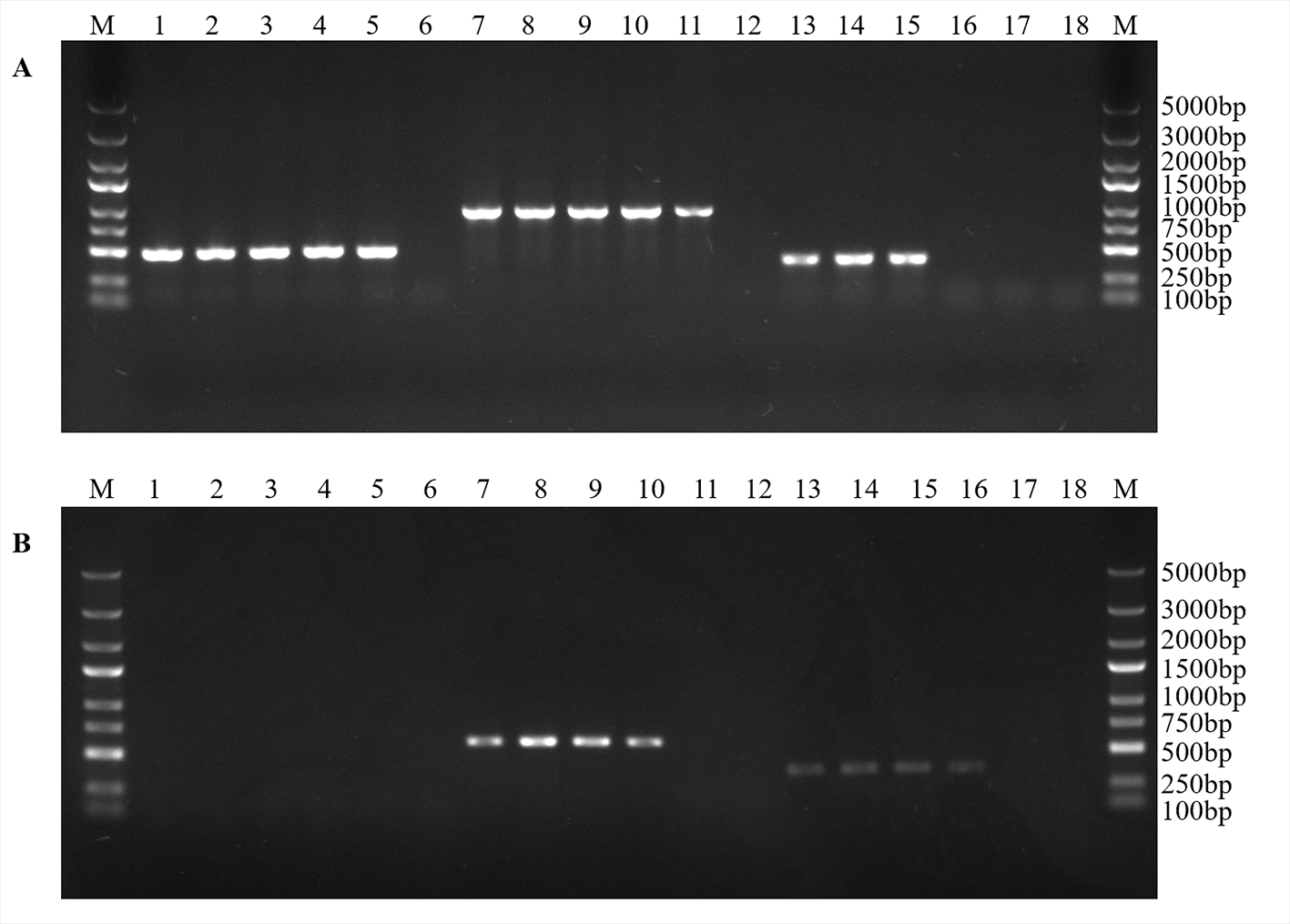

Supplement: Supplementary file 2 [file Image_2.TIF]

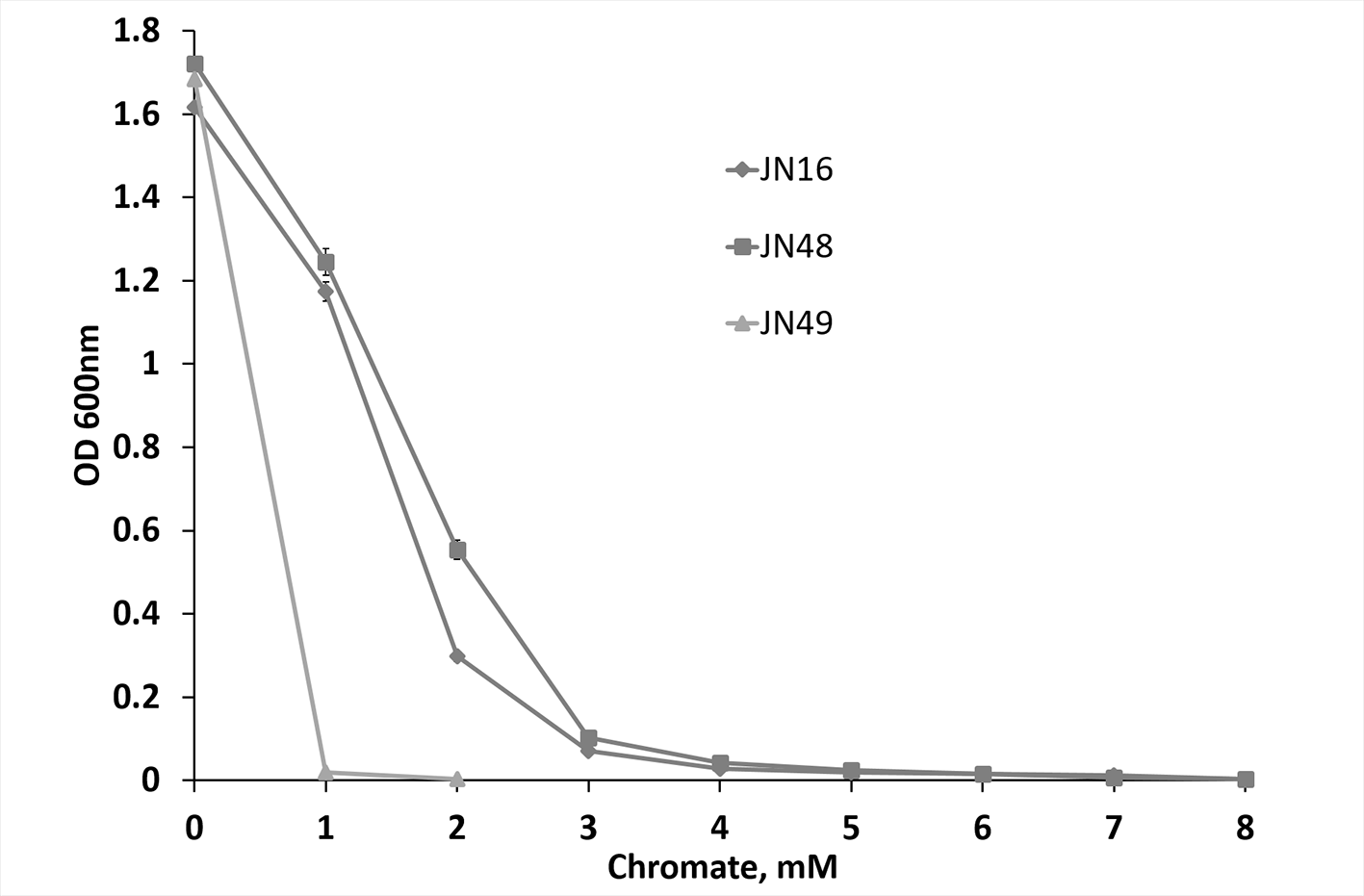

Supplement: Supplementary file 3 [file Image_3.TIF]

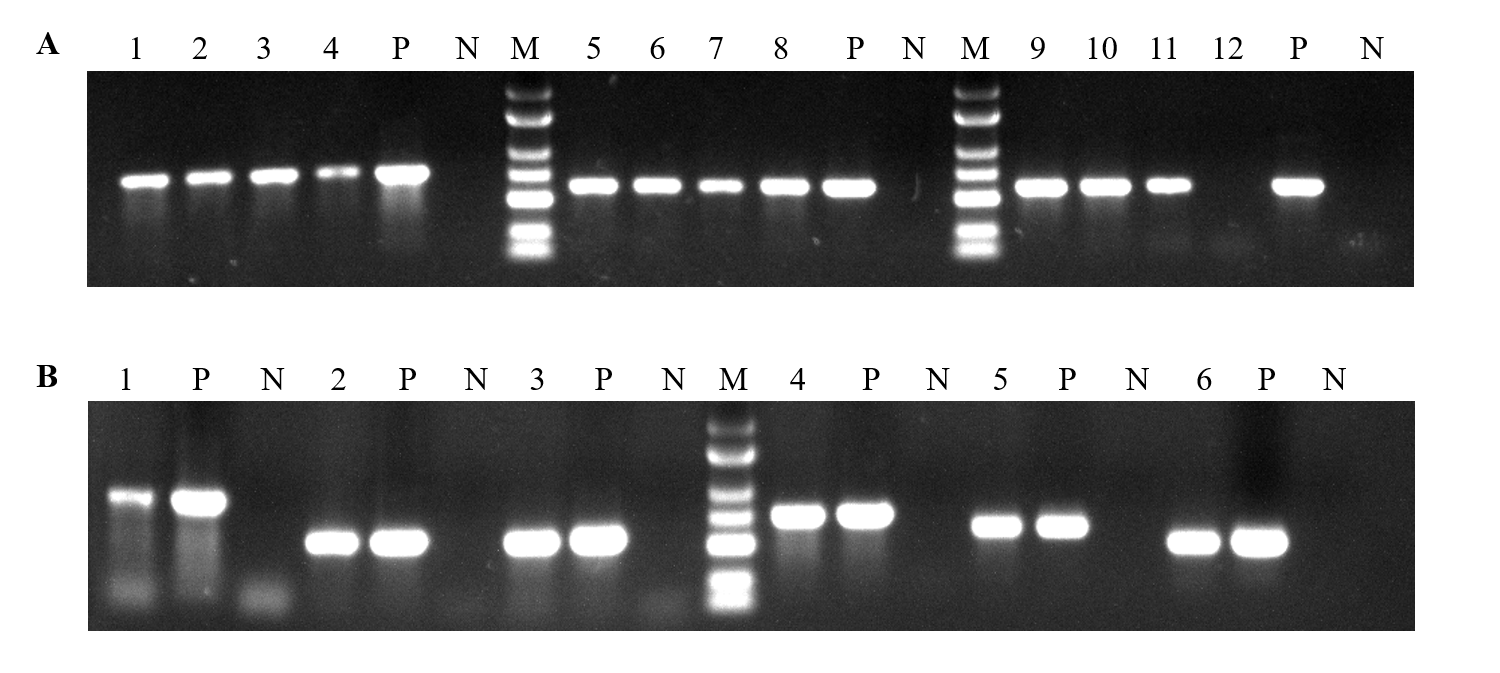

Supplement: Supplementary file 4 [file Image_4.TIF]
