# Supplementary material for: Identification and Characterization of New Resistance-Conferring SGI1s (Salmonella Genomic Island 1) in Proteus mirabilis
Source: Front Microbiol. 2018 Dec 19;9:3172. doi: 10.3389/fmicb.2018.03172 (PMC6305713; doi:10.3389/fmicb.2018.03172)
Supplement: Supplementary file 5 [file Data_Sheet_1.PDF]

***Supplementary Material:***

# Identification and characterization of new resistance-conferring SGI1s (*Salmonella* genomic island 1) in *Proteus mirabilis*

Luyao Bie [1], Meng Fang [1], Zhiqiang Li [2], Mingyu Wang[1*] and Hai Xu[1*]

[1] State Key Laboratory of Microbial Technology, Microbial Technology Research Institute, Shandong University, Qingdao, China
[2] Advanced Research Center for Optics, Shandong University, Qingdao, China

* Correspondence:
Mingyu Wang wangmingyu@sdu.edu.cn, Hai Xu haixu@sdu.edu.cn.

# 1 SUPPLEMENTARY DATA

# 2 SUPPLEMENTARY TABLES AND FIGURES

## 2.1 Figures

**Figure S1.** Model of the site-specific integration and excision of SGI1. Black arrows represent chromosomal genes of *P. mirabilis*. SGI1 is represented in grey rectangle. SGI1 integrates specifically at the 3' end of the chromosomal *trmE* gene (*attB*). The direct repeats (DR-L/DR-R) are formed by the recombination between the chromosomal *attB* site and the SGI1 *attP* site. Primers (circ1/circ2) used for detection of the circular SGI1 form are indicated by horizontal arrows.

**Figure S2.** Detection of SGI1 and circular extrachromosomal form of SGI1, **(A)** Lane1-6, left junction by PmLJ1/LJR1(JN16, JN29, JN47, JN40, JN48, N); Lane7-12, S026 (JN16, JN29, JN47, JN40, JN48, N); Lane13-18, right junction by 104-RJ/PmRJ1 (JN16, JN29, JN47, JN40, JN48, N), **(B)** Lane1-3, right junction by 104-RJ/hipA-R1 (JN40, JN48, N); Lane 4-6, right junction by 104-RJ/MP-R1 (JN40, JN48, N); Lane7-12, S044 (JN16, JN29, JN47, JN40, JN48, N); Lane13-18, circular extrachromosomal form of SGI1 by circ1/circ2 (JN16, JN29, JN47, JN40, JN48, N). N, negative control; M, marker.

**Figure S3.** Resistance of *P. mirabilis* JN16 and JN48 to chromate.

**Figure S4.** Detection of pR55 and SGI1 in transconjugants. **(A)** Lane 1-4, *parA* of pR55 in *P. mirabilis* (JN29, JN40, JN47, JN48); Lane 5-8, *xis* of SGI1s in transconjugants (SGI1-*Pm*CAU, SGI1-*Pm*ABB, SGI1-*Pm*JN40, SGI1-*Pm*JN48); Lane 9-12, S044 of SGI1s in transconjugants (SGI1-*Pm*CAU, SGI1-*Pm*ABB, SGI1-*Pm*JN40, SGI1-*Pm*JN48). **(B)** Lane 1-3, *orf4*, *orf16*, and *orf17* of SGI1-*Pm*JN40; Lane 4-6, PMI3015, PMI3046, and *chrA* of SGI1-*Pm*JN48. P, positive control; N, negative control; M, marker (2000bp, 1500bp, 1000bp, 750bp, 500bp, 250bp, 100bp).

## 2.2 Tables

**Table S1.** Primers used for PCR in this study.

| Primer | Sequence (5′-3′) | Target gene | Reference |
|---|---|---|---|
| **Detection and location of SGI1** | | | |
| PmLJ1 | ACACCTACAACAAGGCTATC | *thdF* | (Boyd, 2007) |
| LJ-R1 | AGTTCTAAAGGTTCGTAGTCG | *int* | (Boyd, 2001) |
| 104-RJ | TGACGAGCTGAAGCGAATTG | S044 | (Boyd, 2001) |
| PmRJ1 | GATGCACACTGAGTTGATAG | *hipB* | (Boyd, 2007) |

| | | | |
|---|---|---|---|
| hipA-R1 | GCCGCTAATAGGTCAAAGG | *hipA* | (Bi, 2011) |
| MP-R1 | CCCAAATGGCAGAAAGAAT | PMI3124 | (Bi, 2011) |
| S026-F | TGGCTACTGCGGAACAAC | S026 | this study |
| S026-R | TACCTGACTGCCTTCTAG | | |
| S044-F | AGCATTTCCGATGATGTG | S044 | this study |
| S044-R | TTCTCCCTGATCTTCATC | | |
| **Mapping of the SGI1 backbones** | | | |
| intSGI1-F | GGTATCAGTAAACAAGCG | *int*-S005 | (Siebor and Neuwirth, 2013) |
| S005-outF | CACTCTCTCGAATCATCC | | |
| rep-outR | CGACTTGGCACATGATTG | *rep*-S005 | (Siebor and Neuwirth, 2013) |
| S005-F | CATGACGAGGTTTGTTGC | | |
| S005-R | GTTTAACGATGCGGGATC | S005-S010 | (Siebor and Neuwirth, 2013) |
| S010-F | CGAGCTGAATATAGACGC | | |
| S010-R | CGGATACCGTTGAGGCGA | S010-S011 | (Siebor and Neuwirth, 2013) |
| S011-F | TTATTCCGTCGGTGATGC | | |
| S011-outR | ATACACGAGCCAACAAGC | S011-S014 | (Siebor and Neuwirth, 2013) |
| S014-R | AGATGATCGGACATAGCG | | |
| S014-F | TGAGTCTGTGGCATGAAG | S014-S020 | (Siebor and Neuwirth, 2013) |
| S020-mF | TCTGCGGTTCGAGGAATC | | |
| S020-R | GTTTGAGCGTAGTGGTGC | S020-S024 | (Siebor and Neuwirth, 2013) |
| S024-outF | GGCTCATACCAATACAGC | | |
| S024-R | ATGATCCTGGCATGTTCG | S024-S025 | (Siebor and Neuwirth, 2013) |
| S025-mF | TCTTGGGATCCTGATTGG | | |
| S025-R | CACTCTATTTTCCCACCG | S025-S026 | (Siebor and Neuwirth, 2013) |
| S026-F | TGGCTACTGCGGAACAAC | | |
| **Mapping of the MDR regions** | | | |
| S026-outR | CAACCTGAAGGGCAACAG | S026-*aadA2* | (Siebor and Neuwirth, 2013) |
| aadA2-R | CTCGCCTTTCACAAAGCG | | |
| aadA2-outF | ATAAAACGCCTACCTGCC | *aadA2*-S044 | (Siebor and Neuwirth, 2013) |
| S044-outR | TCGCTCGATAACTCTCTC | | |
| S026-outR | CAACCTGAAGGGCAACAG | S026-*dfrA1* | (Siebor and Neuwirth, 2013) |
| dfrA1-R | ACCCTTTTGCCAGATTTG | | |
| orfC-outF | TGGTTCCTGCTTGATTCG | *orfC*-S044 | (Siebor and Neuwirth, 2013) |
| S044-outR | TCGCTCGATAACTCTCTC | | |
| S026-outR | CAACCTGAAGGGCAACAG | S026-*aacCA5* | (Siebor and Neuwirth, 2013) |
| aacCA5-R | TTTACTCTGCTCAACCGC | | |
| aadA7-outF | CGCCAAAGGATGTTGCTG | *aadA7*-S044 | (Siebor and Neuwirth, 2013) |
| S044-outR | TCGCTCGATAACTCTCTC | | |
| S026-outR | CAACCTGAAGGGCAACAG | S026-PSE1 | (Siebor and Neuwirth, 2013) |
| PSE1-R | CTTATCAGCGCGACTGTG | | |

| | | | |
|---|---|---|---|
| PSE1-outF | TACAGCAGTTGTGTGGAG | *bla*PSE-1-S044 | (Siebor and Neuwirth, 2013) |
| S044-outR | TCGCTCGATAACTCTCTC | | |
| IS1359F | TGGGTCCACCACATTGATAC | IS*1359* | this study |
| IS1359R | AAGGGAGATGGCTAAACGAA | | |
| **Detection of the circular extrachromosomal form of SGI1** | | | |
| circ1 | AGCAAAATCGTGAGAAGGGA | *attP* | (Doublet, 2005) |
| circ2 | TGATGAGACACCTGACGAGC | | |
| **Detection of the plasmid R55** | | | |
| parAF | AAAAGTAATCAGCTTCGCCA | *parA* | this study |
| parAR | TAGCCCACCTTCTCTAATAG | | |
| **Detection of the transconjugants of SGI1s** | | | |
| xisF | TCAGGCGGTAGATGTATG | *xis* | this study |
| xisR | GAAAGTGACTGGCGGAAT | | |
| orf4F | TTTACATAGCGGCGAATC | *orf4* | this study |
| orf4R | TTAGCCAGTTTGAGCACC | | |
| orf16F | AGTGGAAGATGCCCGTAG | *orf16* | this study |
| orf16R | ATGTGGTGAGCGATAAGC | | |
| orf17F | TCCAAAGAGTAAAGCCAGTA | *orf17* | this study |
| orf17R | AGCAGGTAAGGTGCGATG | | |
| 3015F | ACACCACGCTCTGTTCCA | PMI3015 | this study |
| 3015R | TGTTTATTCGCCCTACGC | | |
| 3046F | CTCTAAACGACCACCTAT | PMI3046 | this study |
| 3046R | AAAGTCTCAGATGGCAAG | | |
| chrAF | TAACCCTACCGATGACAGCA | *chrA* | this study |
| chrAR | GATAGCAATCACGCCAACC | | |

## References


Bi, S., Yan, H., Chen, M., Zhang, Z., Shi, L., and Wang, H. (2011). New variant *Salmonella* genomic island 1-U in *Proteus mirabilis* clinical and food isolates from South China. *J. Antimicrob. Chemoth*. 66, 1178-1179. doi: 10.1093/jac/dkr030

Boyd, D. A., Peters, G. A., Cloeckaert, A., Boumedine, K. S., Chaslus-Dancla, E., Imberechts, H., et al. (2001). Complete nucleotide sequence of a 43-kilobase genomic island associated with the multidrug resistance region of *Salmonella enterica* serovar Typhimurium DT104 and its identification in phage type DT120 and serovar Agona. *J. Bacteriol.* 183, 5725-5732. doi: 10.1128/JB.183.19.5725-5732.2001

Boyd, D. A., Shi, X., Hu, Q. H., Ng, L. K., Doublet, B., Cloeckaert, A., et al. (2007). *Salmonella* Genomic Island 1 (SGI1), variant SGI1-I, and new variant SGI1-O in *Proteus mirabilis* clinical and food isolates from China. *Antimicrob. Agents.*

*Chemoth*. 52, 340-344. doi: 10.1128/AAC.00902-07

Doublet, B., Boyd, D. A., Mulvey, M. R., and Cloeckaert, A. (2005). The *Salmonella* genomic island 1 is an integrative mobilizable element. *Mol. Microbiol*. 55, 1911-1924. doi: 10.1111/j.1365-2958.2005.04520.x

Siebor, E., and Neuwirth, C. (2013). Emergence of *Salmonella* genomic island 1 (SGI1) among *Proteus mirabilis* clinical isolates in Dijon, France. *J. Antimicrob. Chemoth*. 68, 1750-1756. doi: 10.1093/jac/dkt100
